# Removal of Emerging Contaminants from Wastewater Streams Using Membrane Bioreactors: A Review

**DOI:** 10.3390/membranes12010060

**Published:** 2021-12-31

**Authors:** Arijit Sengupta, Mahmood Jebur, Mohanad Kamaz, S. Ranil Wickramasinghe

**Affiliations:** 1 Radiochemistry Division, Bhabha Atomic Research Centre, Mumbai 400085, India; arijita@barc.gov.in; 2 Ralph E Martin Department of Chemical Engineering, University of Arkansas, Fayetteville, AR 72701, USA; mgjebur@uark.edu; 3 Department of Chemical Engineering, Tikrit University, Tikrit 34001, Iraq; 4 Ministry of Oil, State Company of Gas Filling and Services, Karbala 56001, Iraq; mohanedkamaz@gmail.com; 5 Department of Chemical and Process Engineering, Faculty of Engineering and Built Environment, Universiti Kebangsaan Malaysia, Bangi 43600, Malaysia

**Keywords:** adsorption, emerging contaminants, membrane bioreactor, rejection, surface modification

## Abstract

Water is a very valuable natural resource. As the demand for water increases the presence of emerging contaminants in wastewater has become a growing concern. This is particularly true when one considers direct reuse of wastewater. Obtaining sufficient removal of emerging contaminants will require determining the level of removal for the various unit operations in the wastewater treatment process. Membrane bioreactors are attractive as they combine an activated sludge process with a membrane separation step. They are frequently used in a wastewater treatment process and can operate at higher solid loadings than conventional activated sludge processes. Determining the level of removal of emerging contaminants in the membrane bioreactor step is, therefore, of great interest. Removal of emerging contaminants could be by adsorption onto the biomass or membrane surface, biotransformation, size exclusion by the membrane, or volatilization. Given the fact that most emerging contaminants are low molecule weight non-volatile compounds, the latter two methods of removal are usually unimportant. However, biotransformation and adsorption onto the biomass are important mechanisms of removal. It will be important to determine if the microorganisms present at given treatment facility are able to remove ECs present in the wastewater.

## 1. Introduction

Emerging contaminants (ECs) can refer to many types of chemicals such as endocrine disrupting compounds (EDCs), fire retardants, therapeutics, personal care or household cleaning products, lawn care and agricultural products. These compounds can bioaccumulate in the food web and can adversely affect human health and the environment [1]. No strict guidelines have been implemented to regulate their concentration in drinking water, agricultural water, the air or in the environment in general due to limited information on their interaction or their actual toxicological impact [2]. A chemical can be considered as an emerging contaminant, when it passes certain criteria as stated below [2]:

1. The compound is associated with detrimental effects on public health.
2. The positive and negative effects of the compounds are well established.
3. These contaminants are generally not regulated.

Based on environmental monitoring data, a variety of ECs including antibiotics, X-ray contrast media, plasticizers, UV filters, lipid regulating drugs, anti-microbial agents, stimulants, insect-repellents, hormones, anti-inflammatory drugs, artificial sweeteners, anti-itching drugs, anti-depressants, and anticonvulsants are commonly found in water bodies, due to unregulated or partially regulated disposal procedures [3,4,5,6,7].

Some household chemicals including sprays, lipsticks, beauty creams, shampoos, and sunscreen are found in wastewater [8,9]. Some of these chemicals are completely or partially miscible in water. The fate and the mobility of these compounds was found to be strongly affected by their physio-chemical characteristics: evaporation, solubility, boiling point, chemical structure, presence of specific chemical functionalities, melting point, and complexation/sorption ability of the materials [8]. The presence of these ECs in water systems leads to their uptake by animals and even in plants [10,11]. Finally, they will enter the human food chain, which can have devastating effects on the eco-system and pose a serious threat to human health [12].

In this review, we begin by describing some of the major classes of ECs. The occurrence of a particular group of ECs depends on the source of the wastewater, municipal, industrial etc. Section 2 discusses sources of ECs in wastewaters. From a practical perspective, it is unlikely a treatment facility needs to remove all ECs. Ideally, future treatment facilities will be designed around the major ECs that are expected or known to be present. Section 3 discusses removal of ECs by membrane bioreactors (MBRs). Various MBR configurations and modes of operation are discussed. Finally, the main parameters that affect the performance of an MBR are discussed.

### 1.1. Types of Emerging Contaminants (ECs)

ECs are broadly classified into the following types depending upon their origin [13,14].

1. Pharmaceuticals
2. Personal care products
3. Pesticides
4. Endocrine disrupting compounds (EDCs)

#### 1.1.1. Pharmaceuticals

ECs of pharmaceutical origin can be subdivided into the following categories: (i) anti-inflammatories and analgesics (paracetamol, acetylsalicylic generation acid, ibuprofen, and diclofenac); (ii) antidepressants (benzodiaze-pines); (iii) anti-epileptics (carbamazepine); (iv) lipid-lowering drugs (fibrates); (v) *β*-blockers (atenolol, propranolol, and metoprolol); (vi) anti-ulcer drugs and antihistamines (ranitidine and famotidine); (vii) antibiotics (tetracyclines, macrolides, *β*-lactams, penicillin, quinolones, sulfonamides, fluoroquinolones, chloramphenicol and imidazole derivatives); (viii) other substances (cocaine, barbiturates, methadone, amphetamines, opiates, heroin, and other narcotics) [4,5,15].

**Figure 1:**
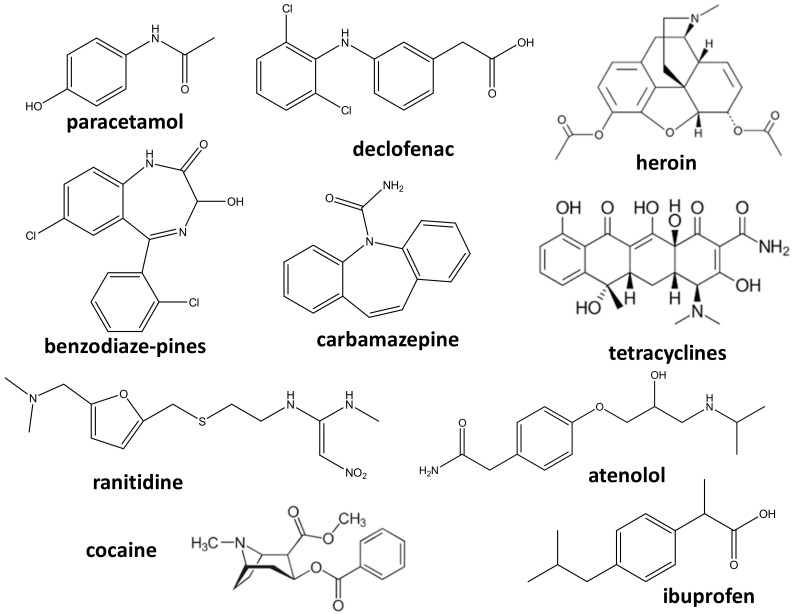
Examples of the pharmaceuticals considered as emerging contaminants (ECs).

Figure 1 shows a few examples of pharmaceutical products considered as ECs. Therapeutic ECs having pharmacological activity and degradation resistance will remain in an aquatic system and could adversely affect the ecology of aquatic microorganisms, fish, aquatic plants, and other aquatic living organisms [16]. This in turn has a negative impact on the health of humans and other living beings, who consume the contaminated water or aquatic plants/animals. The major characteristics, which are responsible for differentiating ECs of pharmaceutical origin from those obtained from other industrial sources are as follows [16]:

- Pharmaceutical ECs are widely varied in terms of molecular weight, structure, functionality, shape, and chemical nature.
- Pharmaceutical ECs are polar, lipophilic molecules with multiple ionizable groups, partially water soluble and the degree of ionization is highly influenced by the chemical nature of the surroundings.
- Pharmaceutical ECs can be persistent for a year (erythromycin, cyclophosphamide, naproxen, and sulfamethoxazole or more (clofibric acid, etc.) and can be a particular concern due to accumulation in humans.
- After administration, the pharmaceutical ECs can be modified chemically during absorption, bio-distribution, and are subjected to metabolic reactions and hence the modified materials may show entirely different chemical/biological effects.

These materials can be excreted without any chemical transformation. If they are metabolically transformed in the body, either they are subjected to biochemical reactions like hydrolysis, oxidation/reduction, alkylation etc. or there could be formation of sulfate conjugates or glucuronide. More polar compounds which are more hydrophilic can enter the environment in two ways: by inclusion in normal rubbish tips, and through feces or urine after their consumption by humans and animals [16,17].

A high death rate due to renal failure has been reported in vultures in India and Pakistan by diclofenac, an analgesic. This analgesic has also been reported to result in renal failure in fish and other aquatic animals [18]. Ibuprofen was found to enhance the prevalence of micronuclei in *Oreochromis niloticus* [19]. Antibiotics like tetracyclines and quinolones were found to have multiple coordinating sites and tend to accumulate heavy metals such as Zn, Cu, Cd, which makes the materials even more dangerous for living beings [20]. Long exposure to antibiotics can generate antibiotic resistant bacterial strains [21]. Antineoplastic compounds (anticancer compounds) Cisplatin and 5-fluorouracil are shown to be toxic to aquatic organisms such as the algae *Pseudokirchneriella subcapitata* [22].

#### 1.1.2. Personal Care Products

These chemicals are the result of urbanization. They are mainly cosmetic products (lipstick, nail polish, talcum powder, sunscreen, lotion, make-up kits etc.), engineered hormones, steroids, perfumes, shampoos etc. [23]. Since their main application is on the surface of the human body, they are not subjected to any metabolic bio-chemical transformation. These ECs are mainly found in urban surface water bodies or aqueous wastewater streams originating from the relevant industries. UV filters were reported to exhibit estrogenic activity [2,24]. Personal care products can be hydrolyzed or undergo oxidation reduction reactions inside water bodies, or they can be adsorbed on the sludge/biosolids, mainly on biologically moderated/transformed lipophilic products [24].

#### 1.1.3. Pesticides

Pesticides are used to protect crops from the unwanted microorganism. They generate microbial resistance during long time exposure and can be transformed by the plant metabolites. They can enter the food chain of aquatic animals and plants [25]. Examples include chlorinated phenoxy acid used as common pesticides in agriculture, herbicides on lawns, algicides in paints and coatings, and roof-protection agents in sealants. These compounds are characterized by high polarity [26].

#### 1.1.4. Endocrine Disrupting Compounds

EDCs are hormonally active. Endocrine disrupting agents can lead to cancerous tumors, birth defects, developmental disorders, problems in the reproductive system, brain, immune system etc. [27]. The relationship between exposure and health effects is complex. Linking a particular EDC with a specific health issue is not very clear [28]. However, fetuses and embryos, whose growth and development are highly controlled by the endocrine system, are more vulnerable to exposure to EDCs [29]. Pre-birth exposure can lead to permanent alterations and adult diseases. Certain cancers and uterine malfunctioning/deformation in women can be linked with diethylstilbestrol (DES) exposure in the womb [30,31].

**Figure 2:**
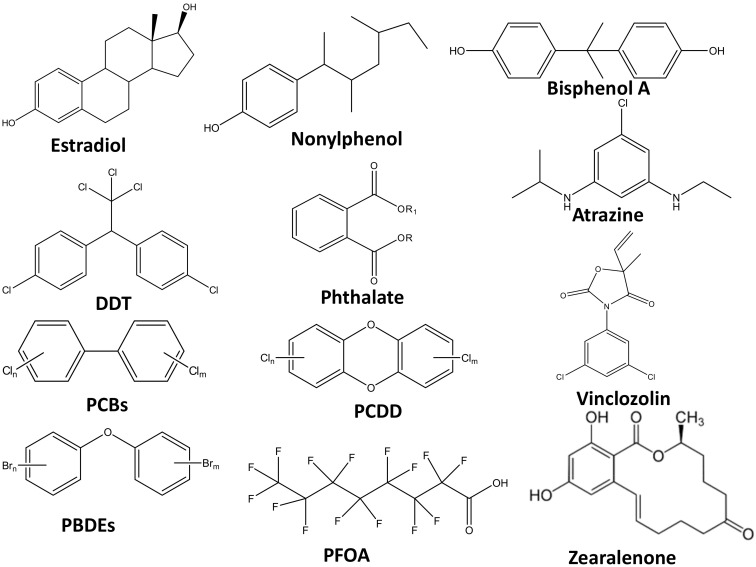
The chemical structures for some endocrine disrupting compounds (EDCs).

Phthalates in pregnant women’s urine can be linked to their male infants in terms of their shorter, female-like anogenital distance and a smaller scrotum and penis [32]. Most of the endocrine disruptors exhibit a U-shaped dose response curve: very low and very high levels have more effects than mid-level exposure. Apart from human beings, other animals were also found to be affected by exposure to endocrine disruptors [33]. The flame retardant, BDE-47, affects the reproductive system and thyroid gland of female rats [34]. These compounds can interfere with the synthesis, secretion, transport, binding, action, and eliminate natural hormones in the body, that are responsible for development, behavior, fertility, and maintenance of normal cell metabolism [35]. Figure 2 shows the chemical structures for some of the EDCs.

##### Xenoestrogens

This EDC is a xenohormone mimicking estrogen. Synthetic xenoestrogens can be found in polychlorinated biphenyls (PCBs), bisphenol A (BPA) and phthalates having estrogenic effects on living beings [36,37]. An example are alkylphenols (APs). Detergents, additives, lubricants, polymers, phenolic resins, thermoplastic elastomers, antioxidants, oil field chemicals, and flame retardants are some of the sources of APs [38,39]. BPA and bisphenol S (BPS) are some of the other EDCs having hydroxyl functionalities. BPA was found in plastic bottles, plastic food containers, dental materials, and the linings of metal food and infant formula cans and can lead to elevation in the rate of diabetes, mammary and prostate cancer, decreased sperm count, reproductive problems, early puberty, obesity, and neurological problems, whereas BPS was found to be in plastics, and household dust exhibiting strong endocrine disruption activity [40,41].

##### Dichlorodiphenyltrichloroethane (DDT)

DDT is one of the best know pesticides having endocrine disrupting ability. DDT exposure has adverse effects on the human reproductive system and can lead to infertility in men, improper development of reproductive systems, and childhood obesity [42].

##### Polychlorinated Biphenyls (PCBs)

This chlorinated EDC can be found in industrial coolants, lubricants, and byproduct in gasoline refining, which can affect liver and thyroid function, enhance childhood obesity, lead to defects in reproductive systems and infertility [43].

##### Polybrominated Diphenyl Ethers (PBDEs)

This neurotoxic EDC was found in plastic cases of televisions and computers, electronics, carpets, lighting, bedding, clothing, car components, foam cushions and other textiles. PBDEs can lead to an imbalance in thyroid hormone resulting in a wide range of neurological and developmental deficits and learning disabilities [44].

##### Phthalates

Soft toys, flooring, medical equipment, cosmetics, and air fresheners are some of the common sources through which there is appreciable chance of phthalates exposure to human beings. Bis(2-ethylhexyl) phthalate (DEHP), used in medical tubing, catheters, and blood bags, can have harmful effects on the sexual development in male infants. Phthalate exposure can also result in masculine neurological development disruption. Perfluorooctanoic acids (PFOAs), polychlorinated dibenzo-dioxins (PCDDs), polychlorinated furans (PCFs), polycyclic aromatic hydrocarbons (PAHs), phenol derivatives, atrazine, vinclozolin, 17-α ethinylestradiol, and zearalenone are examples of some of the well-known endocrine disrupting materials [45].

## 2. Occurrence of ECs in Wastewater Streams and Their Major Source

### 2.1. Industrial Sources

The pharmaceutical and biomedical industries generate significant quantities of waste streams containing bio-active compounds, which include different medicines (antibiotics, analgesic, antidepressant etc.), their unused precursors and side products during manufacture. The bioactivity of the therapeutic compounds may be well known for side products, intermediates, and unused precursors. There is often less information available regarding their chemical, physiological and anti-microbial activities. Apart from that, some illegal drugs, synthetic/natural hormones, steroids, and other ECs can be present in waste streams. Hence careful control and their environmental impact must be investigated before their actual disposal. If required, the waste streams must be properly treated before disposal [46].

Another potential industrial source of ECs is the textile industry. Chemically toxic dyes, their degradation products, and other related hazardous materials are expected in such industrial waste [47]. The food and beverage industries are some of the potential sources of artificial sweeteners, food color, and some of the nanoparticles from industrial waste, which are potential ECs resulting from urbanization [47].

Perchlorinated compounds are extensively used as dirt, or grease repellent coatings and sprays for leather, textile, and in polytetrafluoroethylene (PTFE) (Teflon) non-stick cookware. They are of high environmental risk and a threat to microorganisms when accumulated to an appreciable extent [48]. Flame retardants from plastics, textiles, furnishing foams for television, computers other electronic and sofas etc. are some of the ECs generated industrially as an outcome of globalization [49]. Organic solvents, herbicides, fungicides, insecticides, plant growth regulators, bactericides, and defoliants are also industry originated ECs, that can be added to the environment and surface water and ultimately into the food chain [50].

### 2.2. Municipal Sources

Municipal wastewaters are another source of ECs. The use products such as nail polish, lipstick, sunscreens, lotions, mosquito repellants etc. often result in their entry into municipal wastewater [51]. Other ECs, such as drugs, artificial sweeteners, etc. can be transformed in the human body. They can be excreted through urine, which ultimately leads to their introduction into municipal wastewater [52]. Some ECs can be generated from agricultural activities and are found in agricultural wastewaters. They can also find their way into municipal wastewater [53]. Clearly specific limits on ECs in treated water are required. Strict laws must be enforced depending on the potential risk and hazards associated with different classes of ECs. Removal of ECs from municipal wastewaters will be required [54]. Table 1 summarizes various side effects of different groups of ECs. 

## 3. Removal of ECs by Membrane Bioreactors (MBRs)

An MBR is a blending of a membrane process (microfiltration, ultrafiltration) and a biological wastewater treatment using activated sludge. In recent years, the MBR has been widely used for municipal and industrial wastewater treatment. Recent publications inidacate the feasibility of using an MBR to remove ECs [67,68,69]. Crespo et al. [70] documented the use of MBRs for removal of anionic ECs from drinking water. Pressure-driven MBRs, gas transfer MBRs and ion exchange MBRs were reported to show promising results for EC removal. Chen et al. [71] have reported BPA removal using an MBR and compared the results with a conventional activated sludge reactor (CASR). Higher volume loading was possible for the MBR compared to the CASR without any compromise on BPA removal efficiency.

Clara et al. [72] reported the separation of eight pharmaceuticals, two polycyclic musk fragrances and nine EDCs using an MBR. A comprehensive comparison was carried out with conventional wastewater treatment technology. The MBR was found to be advantageous compared to conventional systems. The detention of particulate matter in the MBR resulted in a suspension free effluent. For strongly adsorbing materials, total emissions were reported to be slightly lower in MBRs than conventional technology. MBRs exhibited better solids retention time within compact reactor volumes resulting in an improved degradation and intensified process.

Kim et al. [73] used an MBR for the removal of acetaminophen, caffeine, metformin, 2-hydroxy-ibuprofen, paraxanthine, ibuprofen, naproxen, clarithromycin, metformin, atenolol, carbamazepine, trimethoprim triclosan, ciprofloxacin, norfloxacin, triclocarban, metformin, caffeine, ofloxacin, and paraxanthine from different aqueous streams of a wastewater plant. They showed that pharmaceutical and personal care product (PPCP) removal varied from −34% to >99% and 23 PPCPs had more than or equal to 90% removal. The performance of the MBR was reported to decrease with filtration time attributed to deposition/cake formation and pore blocking by rejected species on the membrane surface. Membrane fouling resulted in enhancement in hydraulic resistance or transmembrane pressure. Since for wastewater streams, the nature of chemical contaminants and their compositions, feed acidity and pH vary widely, it is impossible to predict the degree of membrane fouling. Several methods, inlcuding air bubbling, and aeration are used to avoid fouling of the membrane in a MBR [74]:

- Intermittent relaxation in filtration can be used to reduce membrane fouling as during relaxation the materials deposited on the membrane surface can defuse back to the reactor.
- Backwashing of the membrane with distilled water can lead to back flow of the water through membrane. This could result in release of the fouling layer/pore blocking substance.
- Backwashing by air at a specified pressure on the permeate side can cause a build up and release of pressure within a very short period. Air usually does not go through the membrane. This can lead to the release of adsorbed foulants.
- Proprietary anti-fouling products (Nalco’s Membrane Performance Enhancer Technology) can be used to reduce fouling.
- Chemical cleaning can be used for dissolving/removal of the fouling layer.
- Chemically enhanced backwash can also be used for cleaning

Higher solids loading in MBRs compared CASRs enhances the uptake rate of ECs resulting in a better degradation in a finite duration or the need for a smaller reactor volume. In an MBR, 96–99% of COD can be removed. A decrease in floc size was found due to hydrodynamic stress in MBRs, leading to enhancement in the apparent reaction rate [75].

### 3.1. Current MBRs

**Figure 3:**
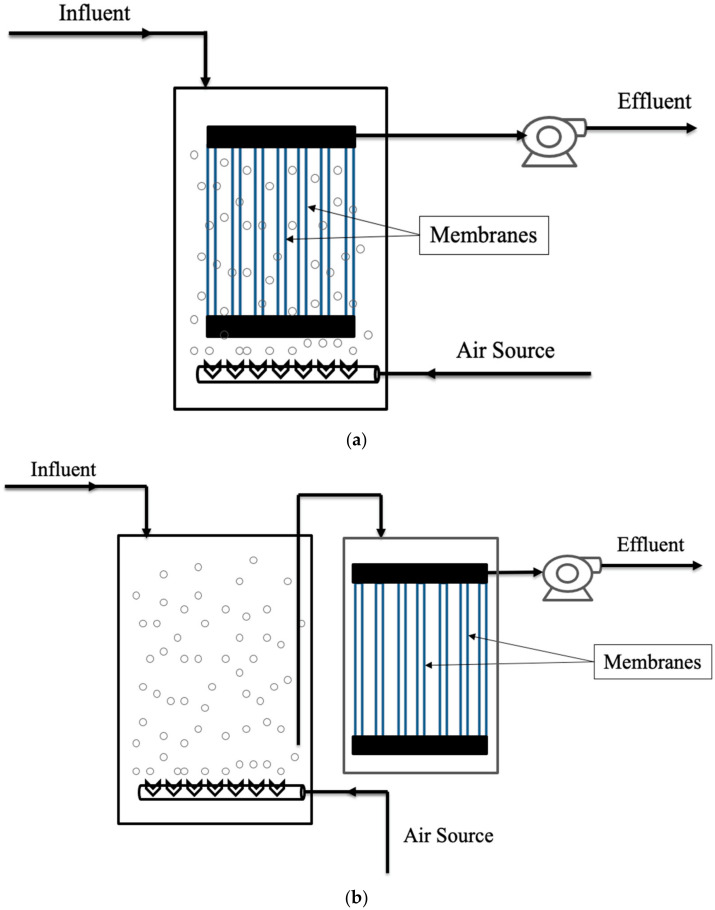
(**a**) Current membrane bioreactors (MBRs); (**b**) first-generation MBRs.

In the current MBR configuration (Figure 3a), flat sheet or tubular or a combination of both membrane modules is placed inside the main bioreactor vessel above the aeration tube [76]. An online backwash system can be used for reducing surface fouling. In a current MBR, the membrane module can also be placed in a separate tank, for which additional aeration needs to be provided to decrease fouling. The energy consumption of the MBR configuration was found to be less compared to other configurations. The biodegradation rate of the ECs was also found to be less [77].

### 3.2. First-Generation MBRs

In the first generation MBR configuration, the membrane modules are outside the aerobic tank, and the aeration system is used to supply oxygen to the microorganisms, which are responsible for the biodegradation as shown in Figure 3b [78]. In general, small-scale high-strength applications are targeted for this type of configuration. This configuration is costlier than the current MBR. The first-generation MBR can handle higher mixed liquor suspended solids (MLSS) concentrations than the conventionl MBR resulting in a compact system associated with easy maintenance, module replacement and cleaning [79].

### 3.3. Hybrid Systems MBRs

Various hybrid and integrated MBR configurations have been explored [80,81,82]. Keerthi et al. [83] have a reported hybrid MBR consisting of integrated electrocoagulation, biological, and microfiltration processes for the treatment of ternary effluents. The results were compared to the current MBR. The hybrid system not only reduced fouling on the membrane surface but also increased the removal efficiency of ECs to more than 90%, which was otherwise 73% for a current MBR. Holloway et al. [84] reported the removal of 20 organic ECs from municipal wastewater using hybrid ultrafiltration-osmotic MBR. Out of 20, 15 ECs were found quantitatively to be removed by this hybrid system. Recently, Li et al. [85] have demonstrated the successful use of a hybrid MBR with nanofiltration (NF) at pilot scale for the treatment of textile wastewater streams to remove toxic dyes and other substances. This MBR-NF process reduces the footprint by 13.7% compared to the existing process. An electrically enhanced MBR has also been reported for the separation of organics, nutrients, and metals [86].

Chon et al. [87] reported a MBR integrated with NF having 210 Da molecular weight cut off revealing a drastic reduction in total nitrogen (TN) and total phosphorous (TP) by nitrification. In this work, the results showed that the surface charge and hydrophobicity of personal care products played a crucial role in their removal. An MBR coupled with an electrocoagulation system has been exploited for the separation of ECs in municipal wastewater [88,89]. Recently, Arcanjo et al. [90] used a hybrid anaerobic osmotic MBR–membrane distillation system to treat municipal sewage to obtain more than 99.9% removal of estrogenic activity.

### 3.4. Aerobic and Anaerobic MBRs

Anaerobic processes are associated with a low-cost treatment, maximum energy recovery without any advanced treatment (low carbon removal, no nutrients removal) [91]. However, membrane-based methods provide the reverse characteristics of advanced treatment and minimum energy recovery. Therefore, a combination of both optimizes the process to make it practical and economically viable. Anaerobic-aerobic MBRs provide smaller footprint compared to the purely aerobic system [92]. They generate methane gas, which can be utilized for different energy recovery processes. They also reduce the cost significantly and generate lower biomass.

Advantages of anaerobic treatment:

- Handles a large variation in organic loading;
- Changes to a non-working mode easily in case of low organic loading;
- Achieves a quick restart and response;
- Converts more than 90% biodegradable organics into biogas compared to aerobic systems;
- Tolerates significant quantities of fats and inorganics.

**Figure 4:**
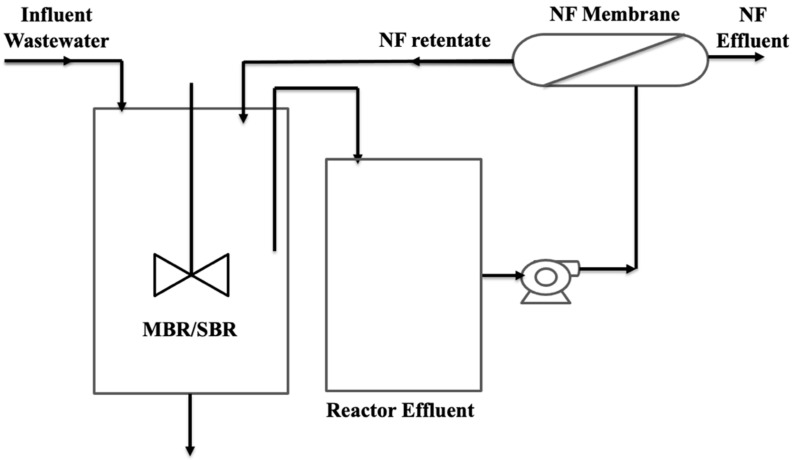
Nanofiltration-coupled membrane bioreactors (aerobic and anaerobic) system.

Sawaya et al. [93] reported the removal of ECs utilizing the membrane biofouling layer, which otherwise was not achievable by microfiltration or ultrafiltration membranes. The anaerobic microbial communities, which are responsible for the biofilm formation on the surface of the membrane played vital roles in such removal. Harb et al. [94] reported the mechanism for modifying the microbial community and their gene expression in organic micropollutants inside aerobic and anaerobic MBRs, as shown in Figure 4.

Pathak et al. [95] recently reported a comparative evaluation of the separation of micropollutants using a MBR and a high retention MBR. Compared to the activated sludge process, a permeate with very low organic content was obtained. Volatilization, size exclusion, electrostatic repulsion or adsorption were reported to influence the processes. Membrane characteristics including pore diameter, molecular weight cut-off (MWCO), surface charge, hydrophilicity/hydrophobicity, surface-solute interactions, and the nature of the feed were found to govern the overall removal mechanism. Sorption, biodegradation, and membrane rejection were reported to separate micropollutants using anaerobic/aerobic MBRs. They reported that hydrophobic, non-ionic ECs are preferentially adsorbed and biodegraded [54].

Kamaz et al. [96] reported the removal of EDCs from wastewater using an aerobic -anaerobic MBR. Almost 20% of the atrazine present was removed by adsorption onto the biomass. Biodegradation of atrazine was reported under aerobic conditions. Abargues et al. [97] reported the separation of alkylphenols: (4-(tert-octyl)) phenol, t-nonylphenol and 4-p-nonylphenol and the hormones (estrone, 17β-estradiol and 17α-ethinylestradiol) using an anaerobic MBR at pilot plant scale using activated sludge wastewater. An Faerobic condition at high sludge retention time (SRT) and hydraulic retention time (HRT) were needed for quantitative degradation of alkylphenols.

Anaerobic conditions were reported to favor the release of alkylphenols and their bioaccumulation. Carbamazepine, acetaminophen, diltiazem, butyl benzyl phthalate, estrone and progesterone removal have been reported by Muz et al. [98] for laboratory-scale demonstration of an anaerobic/aerobic sequencing batch reactor. The Monod model for biodegradation was used for removal of butyl benzyl phthalate, acetaminophen, and progesterone, while low degradation for diltiazem and no degradation but only sorption for carbamazepine were observed. Table 2 depicts the removal of selected ECs via MBRs operated with actual and synthetic wastewater.

## 4. Removal Mechanisms for ECs

### 4.1. Biodegradation

Biodegradation is known as a green technique to control the exposure of ECs [114]. However, the nature of ECs plays a significant role in ascertaining its complete biodegradability. This can be explained in terms of the rate constants of the biodegradation of the corresponding materials. Caffeine, acetaminophen, estradiol, and ibuprofen are some of the examples of ECs, which possess high biodegradation rate constants and hence can be degraded easily into the corresponding elemental precursors losing their bioactivity [115]. However, tetracycline, carbamazepine, and iopamidol possess very low biodegradation constants, thereby leading to incomplete or slow degradation of such compounds [116].

The factors influencing the degradation are redox potential, structural features, microbial diversity, temperature, pH, toxicity of the ECs and primary substrates [117]. Generally, biodegradation/bioremediation of these hazardous materials proceeds through highly specific enzymes [118]. The catalytic amount of such bioactive enzymes is very specific and efficient for transformation of these hazardous materials into their non-active precursors and this transformation can be viable for commercial adaptation, which is known as one of the ‘green’ bioremediations. These enzymes are mostly from oxidoreductase families. Some of the important enzymes are as follows [119]:

- Lignin peroxidase (1,2-bis(3,4-dimethoxyphenyl) propane-1,3-diol;
- Manganese peroxidase (Mn (II): hydrogen-peroxide oxidoreductase;
- Laccases;
- Tyrosinases: o-diphenol;
- Horseradish peroxidase.

#### 4.1.1. Lignin Peroxidase

The oxidative cleavage of the lignin bond in the presence of hydrogen peroxide is the main chemical reaction associated with this enzyme [120]. A wide range of phenolic as well as non-phenolic substances were found to be cleaved by this highly efficient relatively non-specific enzyme [121]. Phanerochaete chrysosporium fungus was the first source of this enzyme however, today it is found to be present in a variety of microorganism including basidiomycetes [122]. Attachment through covalent bond formation, physical entrapment in porous matrices, physisorption/chemisorption and cross linking are some of the modes of immobilization of this enzyme on solid inactive surfaces for biocatalysts [123].

**Figure 5:**
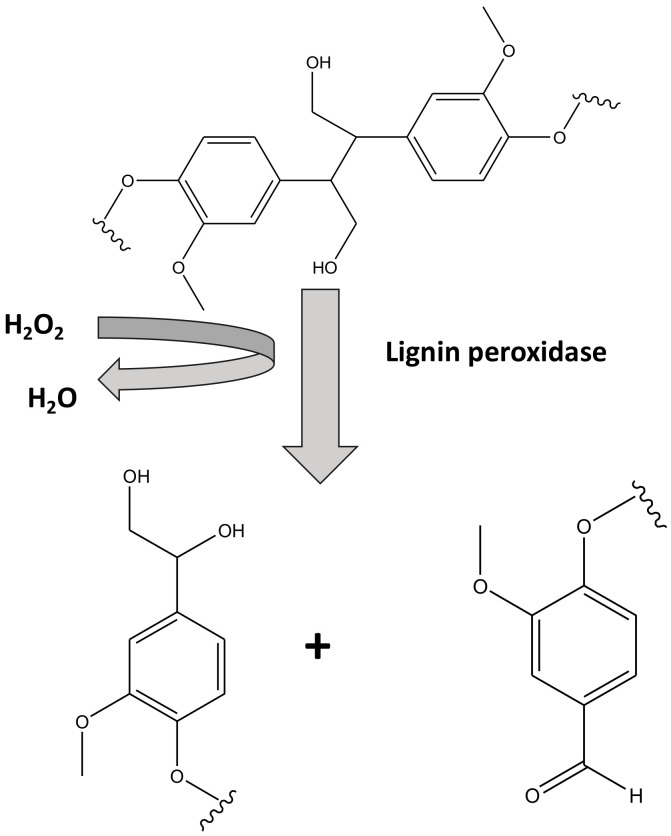
Simplified mechanism of action for lignin peroxidase.

Oliveira et al. [124] and Ran et al. [125] have demonstrated the immobilization of lignin peroxidase through covalent bonding on carbon nanotubes and chitosan with a degradation efficiency of more than 50% and 80%, respectively. Chitosan beads were found to be very good crosslinking support for immobilization of lignin peroxidase obtained from *S. commune* used for the degradation of sandal fix and dyes with efficiencies in the range of ~70–90% [126]. Lignin peroxidase obtained from *G. lucidum* entrapped in Ca-alginate was shown to degrade sandal fix in a highly efficient manner (degradation efficiency ~70–95%) [127]. Nanoporous gold and microporous silica were found to be very good sorbent materials for the lignin peroxidase obtained from *P. chrysosporium* for degradation of dyes like rhodamine blue [128,129,130]. The schematic of the mechanism of biodegradation of methyl orange using lignin peroxidase is shown in Figure 5.

#### 4.1.2. Manganese Peroxidase

**Figure 6:**
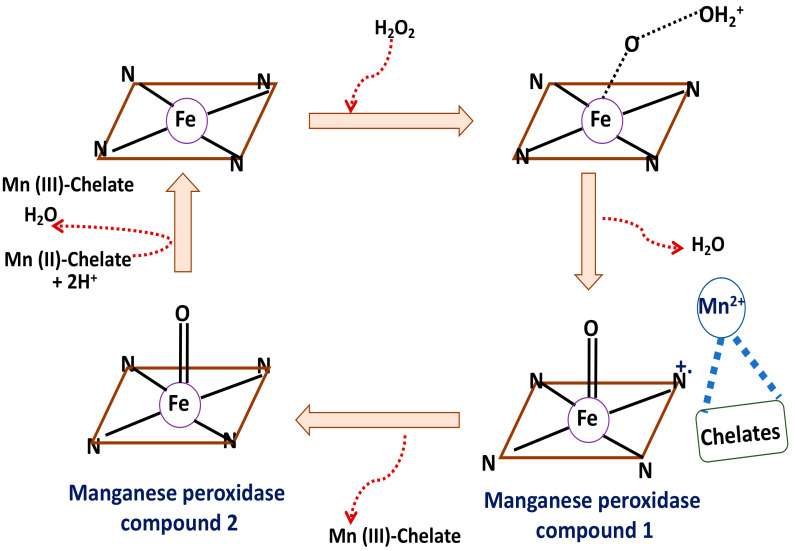
Schematic representation of the simplified mechanism of manganese peroxidase.

A series of irreversible oxidation-reduction reactions in a ping-pong mode has been demonstrated to be the mechanism for manganese peroxidase predominantly following second order rate kinetics. The subsequent electron transfer results in cleavage of the peroxi bonds and formation of H_2_O and the Fe (IV) oxo-porphyrin radical. The next step involves radical quenching through participation of Mn^2+^/Mn^3+^ redox equilibrium releasing a water molecule. Figure 6 schematically represents the mechanism of action of this enzyme.

This enzyme was first reported to be found in *P. chrysosporium*. Toxic, carcinogenic, and mutagenic dyes and monomeric, dimeric as well as polymeric phenolic compounds are the main targets for this enzyme for biodegradation. Bilal et al. [131] have reported the encapsulation of *G. lucidum* to obtain manganese peroxidase on a sol-gel matrix for the biodegradation of the textile effluent from Arzoo, Ayesha, Kalash, Itmad and Crescent with an efficiency of 82–95%. The Ca alginate entrapment of manganese peroxidase has also shown efficient degradation of textile wastes including carcinogenic dyes and their derived compounds [132,133,134,135]. Nano clay was demonstrated as a suitable sorbent for manganese peroxidase immobilization in order to degrade potential aromatic hazards; anthracene, phenanthrene and pyrene [136].

#### 4.1.3. Laccases

These are multi-copper, extranuclear, one electron transfer oxidoreductase divided broadly into three classes, which are found in different bacteria, plant and even varieties of fungus including *Trametes versicolor*, *T. vilosa* or *Cerrena unicolor*. Monophenols, diphenols, polyphenols, monoamines, diamines, N-heterocycles, and phenothiazines are some of the targets for this enzyme [137,138]. Food polymers in the form of proteins and non-starch polysaccharides were found to be crosslinked in the presence of laccases [114]. Formation of covalent bonds, cross-linking, dopamine assisted self-polymerization, physical entrapment into a pore, and adsorption are some of the modes for immobilization of laccases. These laccases were immobilized onto a variety of matrices including copper alginate beads, chitosan, magnetic nanoparticles, chitosan-CeO_2_ microsphere, fibrous polymer, hairy polymer grafted materials, sol-gel matrix, calcium alginate-chitosan beads, TiO_2_-ZrO_2_, TiO_2_-ZrO_2_-SiO_2_ mixed oxide matrices, and multichannel ceramic membranes for degradation of dyes and other textile ECs [139,140,141].

#### 4.1.4. Tyrosinases

These are copper-containing oxidases for melanin production by hydroxylation of mono-phenol to o-diphenol to quinone followed by a series of reactions to melanin [142]. Tyrosinases obtained from different plants, humans, other mammals, and fungi have different structural properties, tissue distribution, and cellular location. The oxidation of phenolic compounds by tyrosinase can lead to the formation of different intermediates having a variety of physio-chemical properties. Crosslinked tyrosinase and laccase aggregates in a hybrid bioreactor were reported to degrade a large number of pharmaceutical products (acetaminophen, naproxen, mefenamic acid, ibuprofen, ketoprofen, indomethacin, tri-methoprim, ciprofloxacin, ofloxacin, caffeine, carbamazepine, bezafibrate, fenofibrate, and atenolol) from municipal wastewater streams in five days [143,144,145]. Edible/non-edible mushrooms have been largely exploited as the source of this enzyme. These enzymes have been immobilized on magnetic iron nano composites, zeolite derivatives, and polyacrylonitrile microspheres to degrade phenols and their derivatives [144,145].

#### 4.1.5. Horseradish Peroxidase

This heme containing enzyme was obtained from the roots of horseradish and extensively used for the oxidation of many phenolic compounds, amines, phenolic acids containing pharmaceuticals, households, dyes, and other industrial ECs [146,147,148,149,150,151]. Immobilization of horseradish peroxidase on Fe_3_O_4_/nanotubes was found to improve the degradation of phenolic compounds [152]. Immobilization on graphene oxide showed almost quantitative removal of phenolic contaminants [153]. Immobilization on glutaraldehyde modified carbon nanosphere showed better pH and temperature stability compared to the free enzyme [149].

### 4.2. Absorption onto the Sludge

Several reports are available in the literature for the removal of ECs using sludge. [154]. The porous structure of the sludge or the biomass present in biotic or abiotic sludge resulted either in entrapment of the hazardous materials through physical adsorption or chemisorption followed by biodegradation. This approach to remove ECs is attractive.

Kamaz et al. [155] have reported the adsorption of Congo red, Remazol Brilliant Blue R and Eriochrome Black Ton activated municipal sludge. A Freundlich isotherm and pseudo-second-order kinetics were reported to be predominating during the sorption of the dyes. A thermodynamic analysis of the sorption process revealed that the processes were spontaneous. Enhancement in entropy leading to spontaneity of sorption was reported for Remazol Brilliant Blue R and Eriochrome Black T. The activated and deactivated sludge under aerobic and anaerobic conditions exhibited different sorption capacities indicating the involvement of different microorganism [155].

Streit et al. [156] have reported the adsorption of ibuprofen, ketoprofen, and paracetamol on effluent treatment plant sludge in the beverage industry. The porous structure with high surface roughness, surface area (642 m^2^ g^−1^), and total pore volume (0.485 cm^3^ g^−1^) was responsible for achieving 145, 105, and 57 mg g^−1^ sorption capacity for these pharmaceutical ECs. Coimbra et al. [157] have reported the adsorption of pharmaceuticals (diclofenac, salicylic acid, ibuprofen and acetaminophen) from municipal wastewater streams using a pulp mill sludge. Although 200 min was required to attain complete equilibrium sorption for all the ECs, their pseudo second-order rate constants followed the trend: salicylic acid > diclofenac > ibuprofen > acetaminophen.

The Sip isotherm was reported to be suitable for explaining the sorption processes with the trend in sorption capacity: diclofenac > ibuprofen ~ acetaminophen > salicylic acid. Removal of 17α-ethinylestradiol, 4-nonylphenol, and carbamazepine in wastewater using an aerobic granular sludge was found to initiate through adsorption followed by degradation with sorption capacity of 16.09 μg/g and 20.05 μg/g, for 17α-ethinylestradiol, and 4-nonylphenol, respectively [158]. Both Langmuir and Freundlich isotherm model has been used to describe the sorption processes. Chiavola et al. [159] reported the adsorption followed by biodegradation of EDCs: BPA, 17α-ethinylestradiol (EE2), estrone(E1) and 17β-estradiol (E2) using activated and inactivated sludge. Pseudo second-order kinetics were reported to be predominate. Temporary inhibition of the biological process was observed at an initial higher concentration of EDCs resulting in a reduction in the mineralization process till the non-inhibiting value of their concentration was reached. They reported the removal of EDCs from wastewater along with simultaneous nitrification.

Recently, activated sludge has been used for 98–99% removal of ibuprofen and paracetamol by adsorption. The kinetics of sorption were found to follow pseudo-first and pseudo-second order models at all concentrations of the pharmaceuticals. Mesoporous biochar obtained from textile mill sludge has been exploited for the removal of ofloxacin pharmaceutical ECs with a sorption capacity of 9.74 mg g^−1^ following a π-π electron donor-acceptor and H bonding mechanism [160]. The sorption processes were spontaneous and exothermic in nature, best described by a pseudo second-order kinetics model and Redlich–Peterson and Freundlich isotherm models through a multilayer sorption process.

Although adsorption of ECs by the sludge is very common and a widely used cost-effective method, several other sorbents have also been evaluated. Some of them are porous materials obtained naturally, some are modified with specific surface functionalities to capture the ECs. Hence, not only efficient separation but also selective separation can be achieved. More than 90% separation of hormones (17α- dihydrouridine, 17α-Estradiol, 17α-ethinylestradiol, progesterone, estriol, and estrone) can be achieved using cyclodextrin coated silica [161]. However, the same sorbent showed only ~75% efficiency for norgestrel hormone.

The antidepressant, fluoxetine, was found to be adsorbed on zeolite, olive stone, sunflower, and walnut shell with sorption capacity in the range of 10 to 44 mg g^−1^ [162]. The same sorbents were also reported to separate nicotinic acid and pharmaceutical compounds, with higher sorption capacity in the range of 57 to 92 mg g^−1^. Avocado seed activated carbon was also reported to be very sorbent materials for sodium diclofenac analgesics with a capacity of 395 mg g^−1^ [163].

Zn based metal-organic frameworks have been reported to adsorb amodiaquine, whereas carbon nanotubes, graphene and its derivatives were also utilized for adsorption of diclofenac, carbamazepine, and ciprofloxacin [164].

### 4.3. Retention by the Membrane

**Figure 7:**
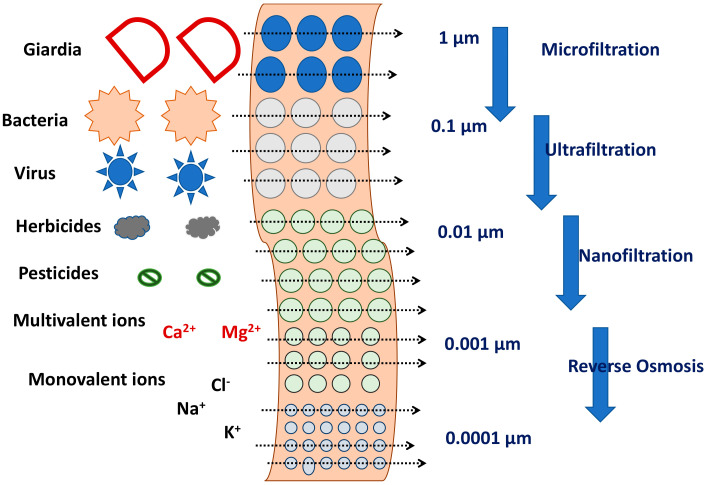
Sieving of ECs through membranes with different pore sizes.

The retention of ECs by the membrane could be by adsorption or size exclusion. Retention by the membrane depends on the membrane pore size and pore size distribution, surface hydrophilicity, morphology, and roughness which in turn are affected by the membrane polymer [165]. Since the ECs possess a wide range of physio-chemical properties, the level of retention by the membrane can be highly variable depending on the specific EC. As indicated in Figure 7, if size exclusion is the main mechanism of retention, then the size of the EC relative to the membrane pore size is very important. If surface adsorption is the main mechanism of retention, the membrane surface interaction with the EC will be more important. Some general observations are as follows.

- Physical sieving can be used for the retention of non-ionic hydrophilic ECs (e.g., paracetamol, caeine, methylparaben);
- Surface interaction and initial adsorption is the major phenomena during retention of hydrophobic non-ionic ECs (e.g., carbamazepine, estrone). It was also reported that, there is a reduction in ECs rejection after the absorption saturation;
- For ECs with charged surface (either positive: propranolol, metoprolol or negative: ibuprofen, naproxen, diclofenac), the retention depends on electrostatic interaction between ECs and membrane materials in combination with sieving.

NF membranes were used for the retention of steroids [166]. Size exclusion and surface adsorption were reported to be the main mechanism of retention. Reverse osmosis (RO) has also been used for the removal of ECs [167]. Depending upon the nature of ECs, 40–100% rejection of ECs can be achieved by NF and RO processes [168,169,170].

## 5. Parameters Affecting the Performance of the MBRs

**Figure 8:**
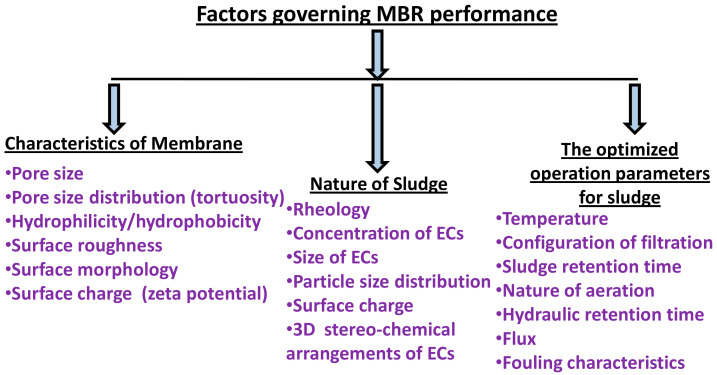
The factors influencing the performance of membrane bioreactor (MBR).

Figure 8 summarizes the main factors that affect the performance of a MBR for removal of ECs: membrane characteristics, nature of the sludge and operating conditions. In fact, it is the interplay of these three groups of variables that will determine the level of EC removal. As seen in Figure 8, there are many factors under each group of variables that affect EC removal.

Membrane morphology and surface properties such as the pore size and pore size distribution, surface roughness, hydrophilicity, and charge on membrane surface influence the performance of the MBR [171]. Rougher surfaces tend to increase interactions with dissolved ECs. ECs present in the wastewater can also influence the MBR performance.

The interaction between the membrane surface and the ECs determines the level of adsorption of the ECs on the membrane surface and their subsequent biodegradation. This affects the degree of membrane fouling. Attractive interactions due to the oppositely charged surface and van der Waal’s interactions can generate a deposition layer on membrane surface. The sludge growth rate (*R_m_*), biodegradation rate (*−R_d_*) and sludge yield (*Y*) can be expressed as follows [172]:

(1) $$R_{m}=(\frac{X_{r}}{SRT}+\frac{dX_{r}}{dt})$$

(2) $$-R_{d}=[\frac{C_{i}-C_{e}}{HRT}+\frac{C_{i}-C_{s}}{SRT}-\frac{dC_{s}}{dt}]$$

(3) $$Y=-\frac{R_{m}}{R_{d}}$$

where, *HRT* is hydraulic retention time, *SRT* is sludge retention time, *X_r_* is volatile suspended solid concentration, *C_i_, C_e_* and *C_s_* are the chemical oxygen demand in influent, effluent, and supernatant, respectively.

## 6. Conclusions and Outlook

ECs can be found in surface water, underground water and in drinking water mainly associated with human activities. Municipal wastewater and industrial effluent discharges are the major sources of these ECs, which include therapeutic as well as illicit drugs, pesticides, herbicides, anti-microbial agents, growth hormones etc. used for agricultural purposes, personal care products, and industrially hazardous substances etc. ECs are often detected at low levels in wastewater effluents. A total of 1800 engineered nanotechnology substances have been recognized as ECs and their in vivo as well as in vitro risk assessment have been carried out [173,174].

Biodegradation of ECs depends strongly on carbon loading, redox conditions, HRT, SRT, and the microbial community composition. Often the specific microbial community that exists is highly sensitive to specific ECs. Thus, it is important to determine families of microorganisms present e.g., by genetic profiling in order to determine the likelihood of degradation of a specific EC. In the case of an MBR, ECs may also be removed by size exclusion although the EC needs to be bigger than the membrane pores.

The precursors hazardous materials originated from pharmaceuticals, industrial, agricultural, and personal care products etc. can undergo different metabolic transformation in humans, plants, and other microorganisms. Biodegradation, chemical oxidation, and other physio-chemical or biological treatment can also lead to the transformed products. The transformed products may or may not have the similar activity either chemically or biologically. The risk associated with these substances must be assessed.

The importance of detecting and removing ECs continues to grow as the demand for water increases the necessity of greater recycling and reuse of wastewater. This is particularly important when one considers direct potable reuse of wastewater. Accumulation of ECs in the treated wastewater is a concern. The total removal of ECs in a wastewater treatment process will depend on the sum of the removal obtained for each unit operation. MBRs are frequently used in wastewater treatment processes. Assessing the level of removal by biotransformation and adsorption will be important. Given the fact the ECs are generally low molecular weight non-volatile compounds, it is unlikely they will be removed by volatilization or size exclusion in an MBR.

## Figures and Tables

**Table 1:** Classifications and side effects of the ECs.

ECs Class	Chemicals	Side Effects	References
Pharmaceuticals	roxithromycin, clarithromycin, and tylosin (antibiotics)	growth inhibition of algae	[55]
	penicillin, sulfonamides, and tetracyclines (antibiotics)	resistance among bacterial pathogens	[56,57]
	acetaminophen, amoxicillin		
	Diclofenac (nonsteroidal anti-inflammatory drug)	renal lesions and gill alterations of fish	[58]
	Gemfibrozil (blood lipid regulator)	growth inhibition of algae	[59]
	caffeine (stimulant drug)	endocrine disruption of goldfish	[60]
	Carbamazepine (antiepileptic drug)	oxidation stress of fish	[61]
Personal care products	preservatives, i.e., parabens (alkyl-hydroxybenzoate) used in in cosmetics, toiletries and even foods	shows weak estrogenic activity	[62]
	disinfectants/antiseptics,.i.e., (triclosan—used in toothpaste, hand soaps, acne cream)	acts as toxic or biocidic agent and cause of microbial resistance	[63,64]
Pesticides	atrazine	endocrine disruptors	[65]
	Acetamiprid		[25]
	chlorinated phenoxy acid herbicide		[26]
EDCs	xenoestrogens (polychlorinated biphenyls (PCBs), Bisphenol A (BPA))	estrogenic effects on living being	[36,37,66]
	dichlorodiphenyltrichloroethane (DDT)	effects in human reproductive systems	[42]
	polychlorinated biphenyls (PCBs)	affect liver and thyroid, enhance childhood obesity, defects in reproductive systems and infertility	[43]
	polybrominated diphenyl ethers (PBDEs)	imbalance in thyroid hormone resulting in a wide range of neurological and developmental deficits, less intelligence and disability in learning	[44]
	phthalates	harmful effects on sexual development in male infants	[45]

**Table 2:** Removal of ECs by MBR systems.

EC	Wastewater Source	Removal of MBR %	Reference
Ketoprofen	Synthetic	90	[99]
Pharmaceuticals	Actual	99	[100]
Steriods	Actual	80	[101]
Sulfamethoxazole	Synthetic	99	[102]
Trimethoprim	Actual	65, 70	[103,104]
4-nonylphenol,	Actual	65, 70	[103,104]
Caffeine	Actual	65, 70	[103,104]
Nonylphenol	Actual	80	[105]
Pesticides	Synthetic	(97–99), (98.5–99)	[3,4]
Acetaminophen	Synthetic	95	[106]
	Actual	100, 100	[65,107]
Amoxicillin	Synthetic	77	[4]
	Actual	100, 100	[65,108]
Atrazine	Synthetic	40, 8	[109,110]
	Actual	<25	[65]
Estrone	Synthetic	>90, 88	[4,111]
	Actual	(95–100), 98	[65,100]
Triclosan	Synthetic	>90	[112]
	Actual	98, 100	[65,113]

## Nomenclature

APs	Alkylphenols
BPA	Bisphenol A
BPS	Bisphenol S
CASR	Conventional activated sludge reactor
DDT	Dichlorodiphenyltrichloroethane
DEHPs	Bis(2-ethylhexyl) phthalate
DES	Diethylstilbestrol
ECs	Emerging contaminants
EDCs	Endocrine disrupting compounds
HRT	Hydraulic retention time
MLSS	Mixed liquor suspended solids
MWCO	Molecular weight cut-off
NF	Nanofiltration
PAHs	Polycyclic aromatic hydrocarbons
PBDEs	Polybrominated diphenyl ethers
PCBs	Polychlorinated biphenyls
PCDDs	Polychlorinated dibenzo-dioxins
PCFs	Polychlorinated furans
PFOAs	Perfluorooctanoic acids
PPCPs	Pharmaceuticals and personal care product
PTFE	Polytetrafluoroethylene
RO	Reverse osmosis
SRT	Sludge retention time
TN	Total nitrogen
TP	Total phosphorous
Equation Symbols	
C_e_	The chemical oxygen demand in effluent	
C_i_	The chemical oxygen demand in influent	
C_s_	The chemical oxygen demand in supernatant	
R_d_	Biodegradation rate	
R_m_	Sludge growth rate	
X_r_	Volatile suspended solid concentration	
Y	Sludge yield	
